# Impact of Low Voltage Threshold Adjustment on Activation Mapping Interpretation for Atrial Tachycardia in Low-Voltage Left Atrium

**DOI:** 10.31083/j.rcm2411329

**Published:** 2023-11-24

**Authors:** Hao Wang, Jindong Chen, Xiaohua Zhuang, Siqi Xi, Tian Gan, Ben He, Liang Zhao

**Affiliations:** ^1^Department of Cardiology, Shanghai Chest Hospital, School of Medicine, Shanghai Jiao Tong University, 200003 Shanghai, China; ^2^Department of Cardiology, Shanghai Pudong Hospital, Fudan University Pudong Medical Center, 201399 Shanghai, China

**Keywords:** atrial tachycardia, high-density mapping, low voltage zone, low voltage threshold adjustment, catheter ablation

## Abstract

**Background:**

The misinterpretation of activation propagation within low voltage zone (LVZ) can complicate atrial tachycardia (AT) mechanism analysis, especially in patients with remodeled atrial substrate. This study investigated the impact of low voltage threshold adjustment (LVTA) on left atrial (LA) tachycardia activation mapping interpretation.

**Methods:**

We identified 55 ATs in 42 patients undergoing catheter ablation for LA tachycardia, with a mean LA voltage of <0.5 mV. Activation mapping of LA or both atria was used to evaluate AT mechanisms before and after LVTA. Patients underwent regular clinic follow-up after the procedure.

**Results:**

Comparing activation mapping before and after LVTA revealed four categories: (1) complete change in AT circuit and ablation design in 9 ATs; (2) an unchanged AT circuit but tailored ablation design in 16 ATs; (3) identification of bystander gaps in 3 ATs; (4) an unchanged AT circuit and ablation design in 27 ATs. Effective ablation, defined as AT termination or circuit change, was obtained in all 9 Type 1 ATs and 15 of 16 Type 2 ATs by targeting the critical area identified by activation mapping after LVTA. After a median follow-up of 16.5 months, the cumulative freedom from AT was 69.3%.

**Conclusions:**

In patients with low LA voltage, conduction propagation hidden within LVZ was not uncommon, but is often excluded from activation mapping. LVTA can uncover this subtle conduction propagation with reliable accuracy, improving the veracity of activation mapping, and helping guide subsequent ablation.

## 1. Introduction

During catheter ablation for atrial tachycardia (AT), activation mapping often detects low left atrial (LA) voltage, especially in patients with remolded atrial substrate or iatrogenic interventions such as cardiac surgery or catheter ablation for atrial fibrillation [1, 2, 3]. These conditions may create extremely low-voltage myocardium, generally leading to exclusion from activation mapping to avoid artifacts into the mapping result [4, 5]. This process can be intricate and sometimes results in misleading or even false mechanisms in detailed mapping for AT, capable of hindering the subsequent ablation process [6].

The presence of potentially viable myocardium within scars may present significant concern, as it may form part of an AT circuit or even the true critical isthmus (CI) [7, 8, 9]. In these cases these mapping points may hold vital information for the accurate diagnosis of AT mechanism [7, 8, 9]. However, due to their extremely low voltage, these points may be hard to distinguish from background noise (BGN), leading to their exclusion from activation mapping [4, 8, 10]. But if BGN suppression is satisfactory, the accuracy of mapping results could be enhanced by including these low-voltage points.

The present study was conducted to investigate the impact of low voltage threshold adjustment (LVTA) on activation mapping results. The focus was on AT analysis and the subsequent ablation strategy in LA tachycardias with low LA voltage. By addressing the low-voltage mapping points, this study aims to offer insights into more precise activation mapping techniques.

## 2. Materials and Methods

### 2.1 Study Population

Our center recruited a consecutive cohort of patients who underwent catheter ablation for AT using the Rhythmia mapping system (Boston Scientific, Marlborough, MA, USA) from October 2019 to July 2022. We reviewed the electrophysiological study (EPS) files and analyzed the LA mapping points’ voltage information. While there is no universally accepted cutoff for low LA, a mean LA voltage <0.5 mV was selected as the threshold value based on prior studies [11, 12]. Only LA tachycardias with a mean LA voltage <0.5 mV, identified by high-density mapping, were included in the study. All patients provided written consent to review and include their medical records. The study was approved by Shanghai Chest Hospital Ethics Committee (IS22041) and conducted in accordance with the Declaration of Helsinki (as revised in 2013).

### 2.2 High-Density Mapping and Ablation Procedure

The ablation and high-density mapping procedure details are described in supplementary materials. Briefly, a decapolar coronary sinus (CS) catheter was inserted via femoral vein access for pacing, using a bipolar CS potential as a stable reference for local activation time. Heparin was used to maintain an active clotting time between 300-350s. An IntellaMap ${\mathrm{Orion}}^{\text{TM}}$ multipolar catheter (Boston Scientific, MA, USA) was used for electroanatomic mapping under the guidance of Rhythmia mapping system. Unipolar and bipolar electrograms were combined to obtain accurate annotation of the local activation time of each bipolar electrogram. For fragmented or multiple potential electrograms, the timing in the surrounding area was used to select potential for timing annotation.

If the atrial wave was preceding in the proximal CS lead, Right atrial (RA) mapping was conducted first. If the atrial wave preceded in distal CS leads or RA mapping results suggested LA-originated AT, LA mapping was performed via transseptal approach. This included cases where (1) a missing total cycle length (CL) of >10%; (2) RA activated in a centrifugal pattern and earliest at RA septum. AT mechanism and critical area were analyzed after activation mapping. This was followed by ablation with an irrigated ablation catheter (Intellatip MIFI, Boston Scientific, MA, USA; 43 ℃, 35W, irrigation rate 12 mL/min).

### 2.3 AT Mechanism Diagnosis

The AT mechanism was diagnosed by analyzing the activation map: (1) the origin of AT (LA, RA, or bi-atrial) was identified by CL coverage or earliest activation site within the atrium/atria and (2) the precise AT circuit was identified via activation propagation. The window of interest was set at the CL value, and the propagation of AT wavefront was visualized with a 5–10 ms window of activation and was advanced along the timescale step by step.

Bi-loop AT was defined as two simultaneous macro-reentrant circuits using a common isthmus. Bi-atrial tachycardia (biAT) was diagnosed if the circuit used both LA and RA through two interatrial connections. The possibility of biAT should be considered when a local breakthrough is observed near interatrial connections including the Bachmann’s bundle, fossa ovalis, posteroinferior interatrial connection, and CS ostium. Epicardia connection mediated AT (epiAT) was diagnosed if the wavefront propagation showed a ‘jump-frog’ pattern with focal activation after bypassing the atrial conduction barrier.

### 2.4 Low Voltage Threshold Adjustment

The Rhythmia system developed an algorithm named ‘Confidence Mask’ to assess the reliability of mapping points. This process included two factors: the amplitude of voltage and annotation time consistency. In order to avoid introducing artifacts into the mapping result, mapping points with voltage lower than set threshold or inconsistent local activation time were excluded from activation mapping, and the corresponding area was colored grey. LVTA was performed only in low-voltage mapping points without annotation time inconsistency, which usually exhibits disordered colors, to avoid yielding confusing results.

To the best of our knowledge, there is no absolute voltage cutoff to guarantee the absence of conduction. The threshold for unexcitable myocardium was limited by the BGN level of the mapping system. The Rhythmia system had a low BGN level of 0.01 mV and used a default low voltage threshold (LVT) value of 0.03 mV [4]. When a grey area was present in LA, especially if the mapping points within LVZ appeared to be uniform in color and related to the surrounding area with propagation, LVT was manually tuned to 0.02 mV and 0.01 mV to include as many points into activation mapping as possible. If inconsistent automatic annotation was observed in a few discrete points, manual annotation was performed. Then AT mechanism was re-analyzed.

### 2.5 Ablation Target and Outcome

After re-identifying the AT mechanism following LVTA, radiofrequency catheter ablation was performed. (1) For macro-reentrant AT and bystander conduction gap, linear ablation targeted the CI, the narrowest part of the circuit with slow conduction. (2) For focal AT, the earliest activation site with fractionated electrogram was targeted or in a linear fashion from the circuit to an anatomical barrier or an area of conduction block.

Effective ablation refers to AT termination with sinus rhythm restoration or AT circuit change, the latter of which included changes in conduction path and activation sequence and could manifest as abrupt and sustained changes in (1) global propagation, (2) local conduction connection or exit without altering AT global propagation, and (3) reversal in conduction sequence without changing circuit path. The ablation endpoint included (1) AT termination and sinus rhythm restoration; (2) subsequent substrate modification targeting potential substrate facilitating other ATs, including bystander conduction gaps, slow conduction zone, or local area with complex fractionated atrial electrogram.

### 2.6 Follow-up

Electrocardiographic monitoring was applied to all patients during hospitalization. And after discharge, patients underwent regular clinic visits and were assessed with 12-lead electrocardiography and 24 h Holter monitoring.

### 2.7 Statistical Analysis

Continuous variables were expressed as mean ± standard deviation if normally distributed or medians (range) if abnormally distributed, and their comparisons were conducted by paired-samples *t*-test or Wilcoxon rank sum test. Event-free survival was estimated by Kaplan-Meier method. A *p <* 0.05 was considered statistically significant. Statistical analysis was performed by SPSS 26.0 (IBM Corp., Armonk, NY, USA).

## 3. Results

### 3.1 Patient population

From October 2019 to July 2022, 235 patients underwent catheter ablation for AT in our center by using Rhythmia mapping system, among which 42 patients were enrolled. Forty patients had histories of iatrogenic intervention which included catheter ablation for atrial fibrillation in 28 patients (66.7%) and cardiac surgery in 12 patients (28.6%). All 12 patients underwent mitral valve replacement following cardiac surgery. Additional procedures accompanying these replacements included tricuspid valve plasty (TVP) in 4 patients, MAZE in 2 patients, TVP plus MAZE IV in 3 patients, and TVP plus aortic valve replacement in 1 patient. The baseline characteristics of the patients are summarized in Table 1.

**Table 1.** **Baseline characteristics**.

Variables (n = 42)			Value
Age, y			68.3 ± 9.1
Male (%)			29 (69.0%)
NYHA			
	I		10 (23.8%)
	II		29 (69.0%)
	III		3 (8.2%)
Hypertension			22 (52.4%)
CAD			6 (14.3%)
Diabetes mellitus			8 (19.0%)
Stroke			3 (7.1%)
RHD			10 (23.8%)
Cardiac intervention history			
	RFCA		28 (66.7%)
	Cardiac surgery		12 (28.6%)
	None		2 (4.3%)
BNP, pg/mL (median)			113 (20–664)
Echocardiography			
	LVESD (mm)		30.2 ± 4.9
	LVEDD (mm)		46.5 ± 3.6
	LAD (mm)		44.1 ± 5.2
	LVEF (%)		60.1 ± 8.7
	Mitral regurgitation		
		None	16 (38.1%)
		Mild	16 (38.1%)
		Moderate	10 (23.8%)
	Aortic regurgitation		
		None	30 (71.4%)
		Mild	12 (28.6%)
	Tricuspid regurgitation		
		None	12 (28.6%)
		Mild	17 (40.5%)
		Moderate	13 (30.9%)

Data are presented as mean ± SD or median. BNP, brain natriuretic peptide; CAD, coronary artery disease; LVESD, left ventricular end-systolic diameter; LVEF, left ventricular ejection fraction; LVEDD, left ventricular end-diastolic diameter; NYHA, New York heart association; RHD, rheumatic heart disease; RFCA, radiofrequency catheter ablation; LAD, left atrial diameter.

### 3.2 AT Mapping Results and AT Mechanisms

In total, 55 ATs were identified, with 13 patients having multiple ATs. Of these, 36 ATs underwent LA mapping, which was completed in an average time of 10.9 ± 3.4 minutes. The remaining 19 ATs were mapped with both LA and RA, with an average time of 16.8 ± 2.7 minutes. In terms of mapping points, the mean numbers were 11047.0 ± 4652.6 for LA and 5629.8 ± 1265.7 for RA. The mean total CL was 248.1 ± 52.8 ms, and manual annotation was performed in only 33 points in 5 AT maps.

AT mechanisms identified included 53 macro-reentrant ATs and 2 focal ATs. The macro-reentrant ATs were further divided into (1) 6 biATs; (2) 11 epiATs, with epicardial connections including Marshall ligament in 7 ATs, LA anterior wall lesion in 2 ATs, and LA inferior wall in 2 ATs; and (3) 36 endocardial ATs, including 10 bi-loop ATs and 26 single-loop ATs. The locations of CI included LA anterior wall in 21 ATs, LA posterior wall in 5 ATs, LA roof in 7 ATs, LA inferior wall in 4 ATs, LA lateral wall including mitral isthmus in 18 ATs, LA septum in 1 AT, and CS in 1 AT.

### 3.3 The Impact of LVTA on LVZ and AT Mechanism Analysis

**Fig. 1.**
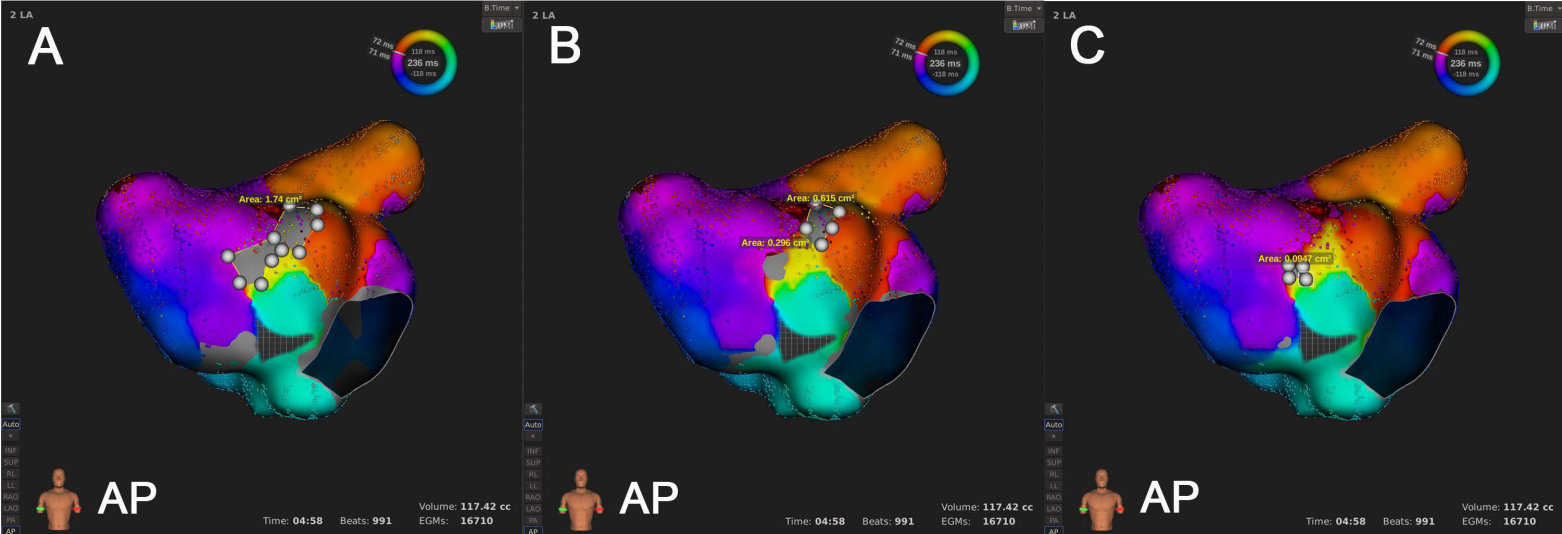
**The impact of low voltage threshold adjustment (LVTA) on low voltage zone (LVZ) in the same map**. As the low voltage threshold (LVT) was adjusted from 0.03 mV (A) to 0.02 mV (B) and 0.01 mV (C), the area of the LVZ on left atrial (LA) anterior wall gradually decreased. Consequently, more points in this area were assigned colors and were subsequently included in activation mapping. The white dots were used to outline the LVZ areas (represented in grey), and these areas were manually measured by the mapping system.

At default LVT (0.03 mV), the spatial relationship between LVZ (grey area) and AT circuit or CI was categorized as follows: (1) away from AT circuit, (2) adjacent to CI, (3) at the corresponding epicardial or endocardial side of CI, and (4) inside the AT circuit. Under this default LVT, the median grey area in the patients was 4.6 ${\mathrm{cm}}^{2}$ (range, 0.36–15.5 ${\mathrm{cm}}^{2}$), and decreased to 2.4 ${\mathrm{cm}}^{2}$ (range, 0–11.9 ${\mathrm{cm}}^{2}$, *p* < 0.001) and 0.3 ${\mathrm{cm}}^{2}$ (range, 0–6.9 ${\mathrm{cm}}^{2}$, *p* < 0.001) when LVT was set to 0.02 mV and 0.01 mV, respectively. In this adjusted setting, some points within previously grey area were assigned colors, allowing for the observation of activation propagation in the newly-colored regions (Fig. 1).

The uncovered propagation impacted AT map interpretations differently, and the ATs were grouped into four categories based on the impact of LVTA (from 0.03 mV to 0.01 mV). The AT mechanism identification and subsequent ablation strategy will be described in the following section.

**Type 1:**

In Type 1, the AT mechanism and CI underwent complete changes, leading to the redesign of the subsequent ablation strategy. This included 9 ATs in 9 patients. The LVZ was located either within the predefined AT circuit or adjacent to the predefined CI. The characteristics of this type of AT have been summarized in Table 2. This Type 1 AT can also be subcategorized into different subtypes, based on the interpretation of activation mapping both before and after LVTA.

**Table 2.** **The results of mapping and ablation of Type 1 AT**.

Patient number	AT number	AT CL (ms)	Mapping point		LVZ area (${\mathrm{cm}}^{2}$)		Subtype	Spatial relation between LVZ and predefined AT circuit/CI	AT mechanism		Effective ablation site	Effect of ablation on AT
			RA	LA	Before LVTA	After LVTA			Before LVTA	After LVTA		
1	1	245	/	8309	3.7	0.5	1.2	LVZ at epicardial side of CI	Epicardial	Endocardial and epicardial	LAAW	Termination
2	2	230	5128	13,581	7.5	0.1	1.3	LVZ inside circuit	Bi-loop	Single-loop	LAAW to MVA	Termination
3	3	246	/	12,378	4.4	0.3	1.2	LVZ at endocardial side of CI	Epicardial	Non-epicardial	MI	Circuit change
4	4	277	/	7050	1.5	0.3	1.2	LVZ at endocardial side of CI	Non-epicardial	Epicardial	LAAW	Termination
5	5	265	/	16,710	1.7	0.1	1.3	LVZ adjacent to circuit	Single-loop	Bi-loop	LAAW to MVA	Termination
6	6	220	4515	11,488	2.4	0.2	1.3	LVZ inside circuit	Single-loop	Bi-loop	LA ridge	Circuit change
7	7	488	4031	9141	8	0.6	1.1	/	uniAT	BiAT	LA septum	Termination
8	8	330	/	6556	2.2	0.2	1.4	LVZ inside circuit	Focal-like	Peri-LIPV	LPV antrum	Circuit change
9	9	235	5206	9745	1	0	1.1	/	Focal-like	BiAT	RPV antrum	Termination

AT, atrial tachycardia; biAT, bi-atrial AT; CI, critical isthmus; CL, cycle length; LA, left atrial; LAAW, left atrial anterior wall; LPV, left pulmonary vein; LVTA, low voltage threshold adjustment; MI, mitral isthmus; LVZ, low voltage zone; MVA, mitral valve annulus; RA, right atrial; LA, left atrial; RPV, right pulmonary vein; uniAT, uni-atrial AT.

**Fig. 2.**
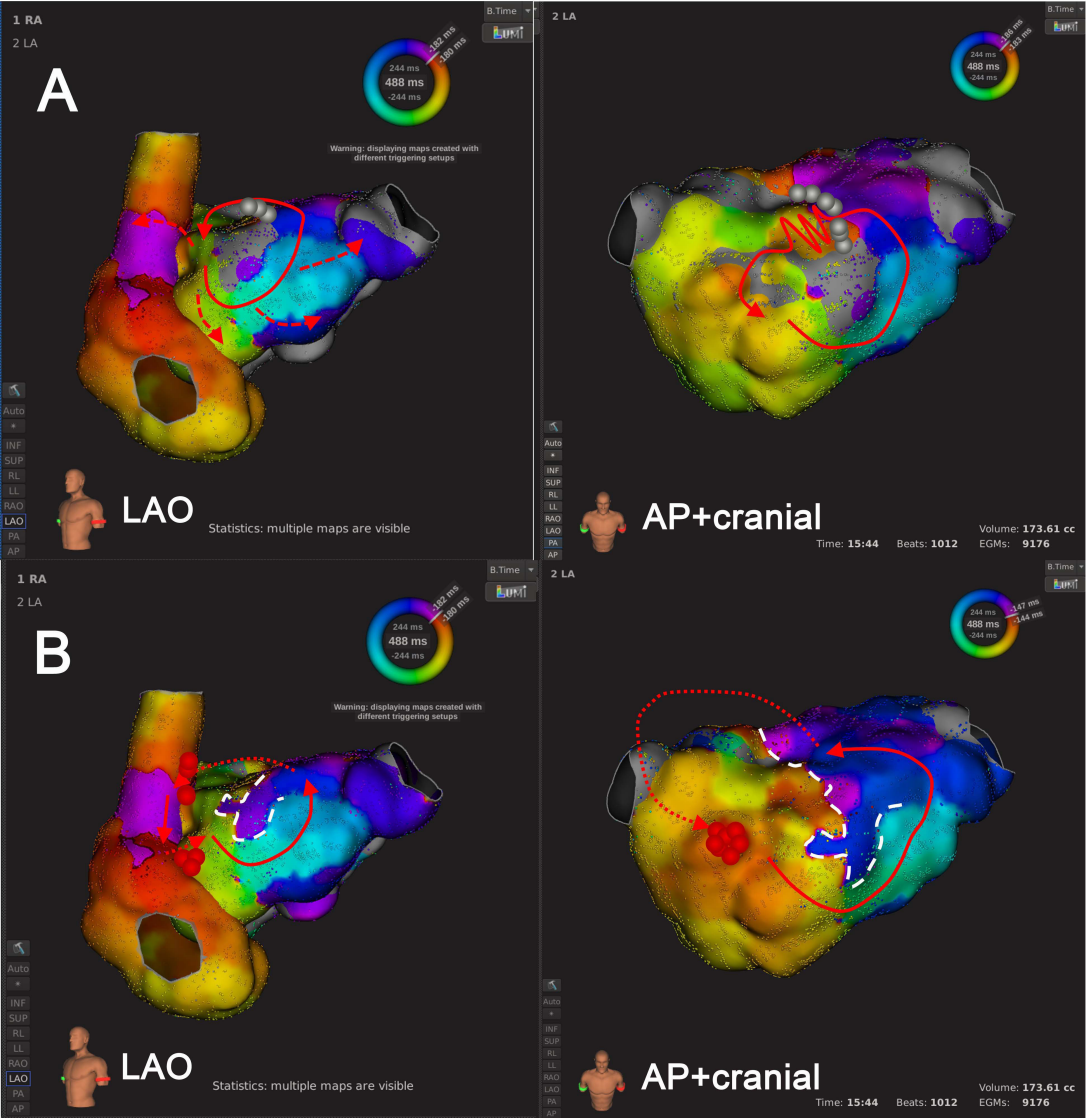
**An example of Type 1.1 mechanism re-identification. **Panel A depicts the scenario at the default LVT of 0.03 mV, where activation mapping suggested a single-loop AT. In this configuration, the wavefront circumnavigated the LVZ at the LA roof, activating both atria centrifugally. Panel B shows that after LVTA (0.01 mV), activation mapping suggested biAT. Furthermore, the wavefront was activated in the LA septum (in an areal pattern), ran around the block line at LA anterior wall, proceeded towards the LA roof, and jumped to right atrium via Bachmann’s bundle. The Solid lines with arrows, the AT circuit as suggested by activation mapping; jagged lines, the wavefront passing through the slow conduction area; dashed red/yellow lines, passive activation propagation; dotted lines, inter-atrial bypasses or epicardial conduction propagation; circle, the origin of focal AT; dashed white lines, conduction block lines; grey dots: planned ablation strategy before LVTA; red dots: effective ablation strategy after LVTA (similarly hereinafter). Abbreviations: AT, atrial tachycardia; biAT, bi-atrial tachycardia; LA, left atrium; LVT, low voltage threshold; LVTA, low voltage threshold adjustment; LVZ, low voltage zone.

**Fig. 3.**
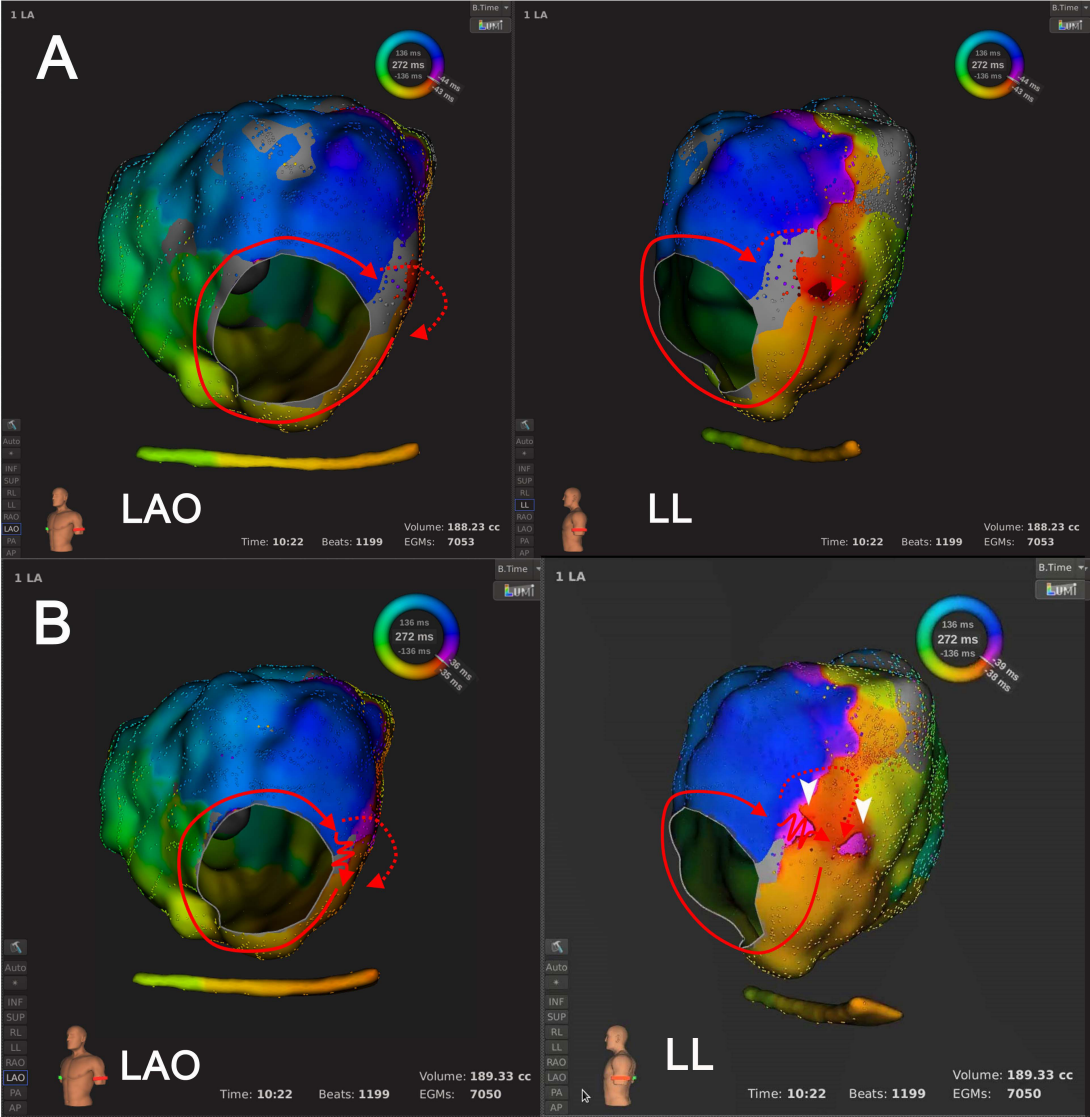
**An example of Type 1.2 mechanism re-identification**. In panel A, at the default LVT of 0.03 mV, activation mapping indicated epiAT and the wavefront circulated around the MA clockwise before jumping across the LVZ at the MI via an epicardial connection. In panel B, following LVTA (LVT adjustment to 0.01 mV), activation mapping suggested clockwise peri-mitral AT passing MI via both endocardium and epicardial connections simultaneously. White arrows indicated simultaneous endocardial and epicardial connections. Abbreviations: AT, atrial tachycardia; epiAT, epicardium-mediated atrial tachycardia; LA, left atrium; LVT, low voltage threshold; LVTA, low voltage threshold adjustment; LVZ, low voltage zone; MA, mitral annulus; MI, mitral isthmus.

**Fig. 4.**
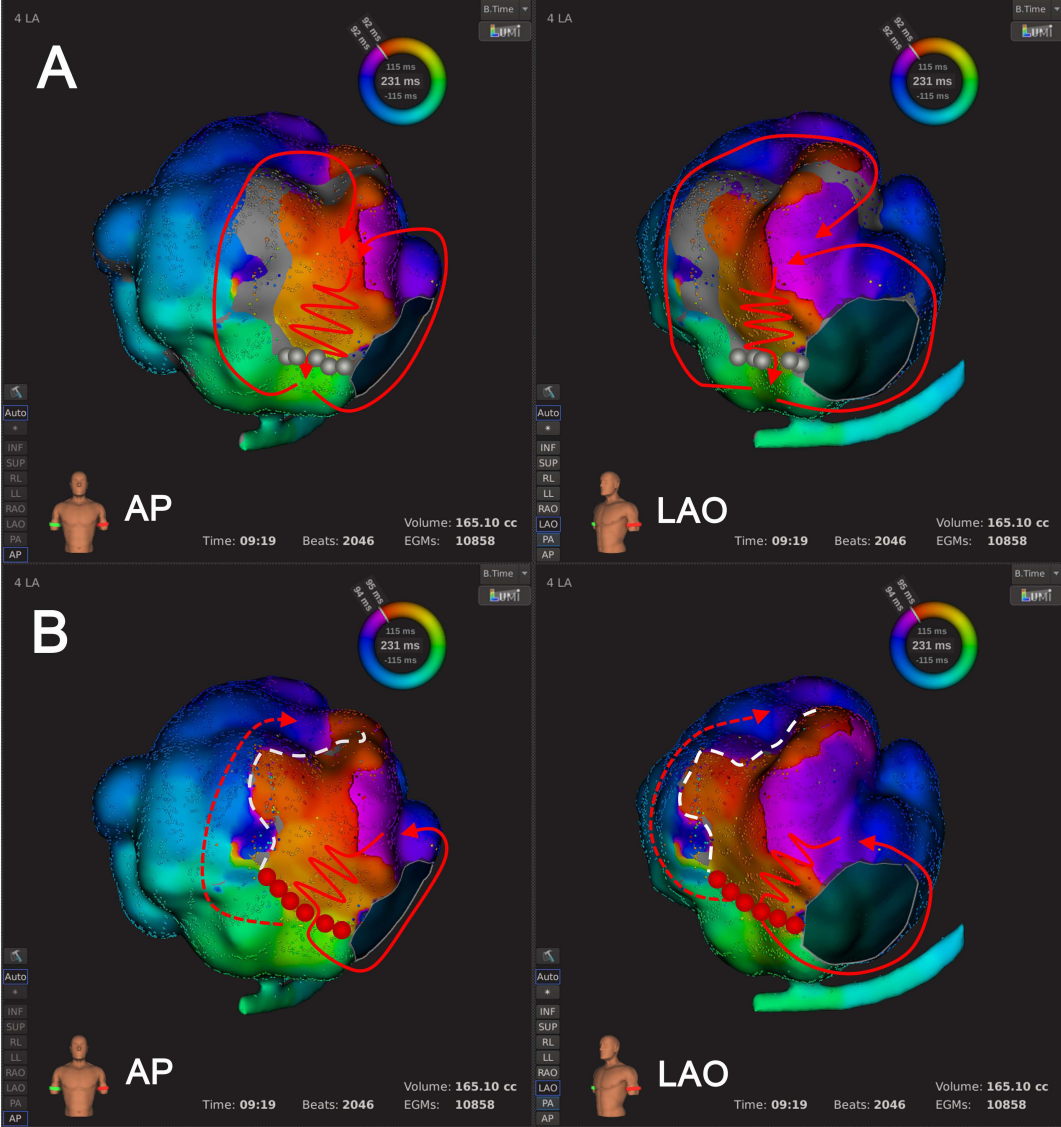
**An example of Type 1.3 mechanism re-identification**. In panel A, at the default LVT of 0.03 mV, activation mapping suggested a bi-loop AT, where the activation wavefront circulated around both the MA and LVZ at LA anterior wall simultaneously, utilizing a shared corridor between the MA and LVZ. In Panel B, following LVTA (0.01 mV), a complete conduction block line across LA anterior wall was revealed within LVZ. Activation mapping suggested single-loop counter-clockwise peri-mitral AT. Abbreviations: AT, atrial tachycardia; LA, left atrium; LVT, low voltage threshold; LVTA, low voltage threshold adjustment; LVZ, low voltage zone; MA, mitral annulus.

**Fig. 5.**
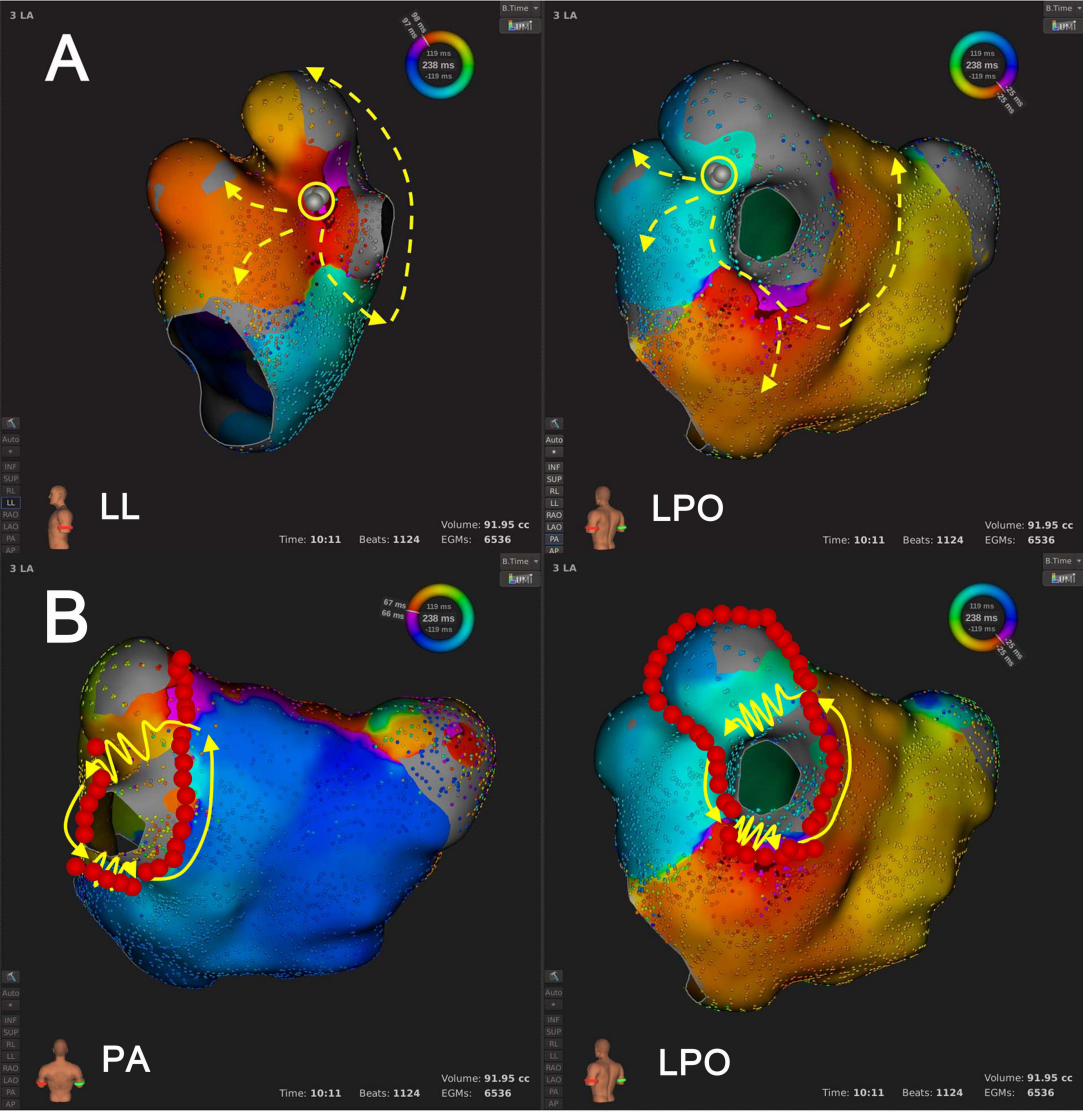
**An example of Type 1.4 mechanism re-identification**. Panel A shows that at the default LVT of 0.03 mV, the LVZ was identified at the left pulmonary ridge. Activation mapping suggested focal AT originating from the left pulmonary ridge. In Panel B, after LVTA (0.01 mV), the activation mapping suggested single-loop reentrant AT, facilitated via two conduction gaps on the pre-ablation line. Abbreviations: AT, atrial tachycardia; LA, left atrium; LVT, low voltage threshold; LVTA, low voltage threshold adjustment; LVZ, low voltage zone.

(1) Type 1.1 consists of uniAT/ biAT, including 2 ATs. The LVZ was located adjacent to inter-atrial connections including LA anterior wall (Bachmann’s bundle branches) and right pulmonary vein antrum (posterior interatrial connections) (Fig. 2, **Supplementary Videos 1** and **2**). (2) Type 1.2 is characterized by epiAT/ non-epiAT, including 3 ATs. The LVZ was found adjacent to the LA roof corresponding to the distribution of Bachmann’s bundle branches or mitral isthmus (including Marshall ligament and CS) (Fig. 3, **Supplementary Videos 3** and **4**). (3) Type 1.3 consists of a single-loop AT/ bi-loop AT, and includes 3ATs. The LVZ was located adjacent to the predefined circuit (Fig. 4, **Supplementary Videos 5** and **6**). (4) Finally, Type 1.4 represents a single-loop AT/ focal AT or a single-loop AT (circuit change), including one AT. The mapping result suggested focal AT at default LVT, but changed to a single-loop epiAT after LVTA (Fig. 5, **Supplementary Videos 7** and **8**).

**Type 2:**

**Fig. 6.**
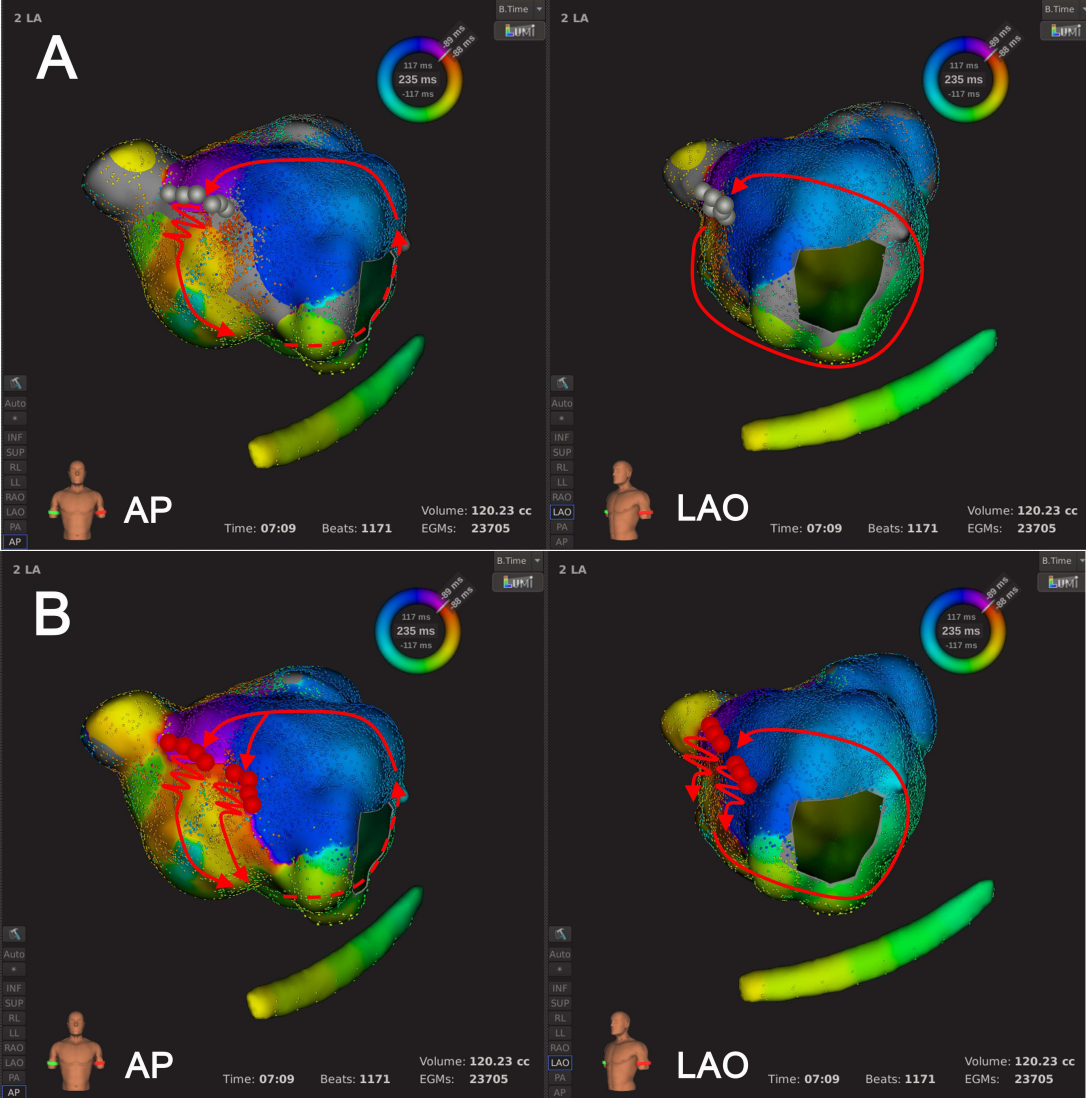
**An example of Type 2 mechanism re-identification**. Panel A shows that at the default LVT of 0.03 mV, the activation mapping suggested counter-clockwise peri-mitral AT. The wavefront crossed the conduction gap within the LVZ, extending from the right superior pulmonary vein to MA. In Panel B, following LVTA (0.01 mV), the AT mechanism remained as a counter-clockwise peri-mitral AT. However, another conduction gap within LVZ was revealed after LVTA, and the wavefront transversed the two conduction gaps almost simultaneously. The dashed red lines indicated AT circuit behind the atrial structure. Abbreviations: AT, atrial tachycardia; LVT, low voltage threshold; LVTA, low voltage threshold adjustment; LVZ, low voltage zone; MA, mitral annulus.

Type 2 cases are characterized by activity following LVTA. While AT circuit and the location of CI remained unchanged, the activation propagation (within the grey area adjacent to predefined CI) was revealed, potentially facilitating activation conduction (Fig. 6, **Supplementary Videos 9** and **10**). In response, the subsequent ablation strategy was tailored by increasing ablation to cover the complete CI, avoiding conduction gap. The extents of CI before and after LVTA were 14.1 ± 4.9 mm and 28.6 ± 7.3 mm, respectively (*p* < 0.001). The width of the conduction corridor within the LVZ measured 14.5 ± 7.0 mm. Type 2 included 16 ATs in 14 patients. The characteristics are summarized in Table 3.

**Table 3.** **The results of mapping and ablation of Type 2 AT**.

Patient number	AT number	AT CL (ms)	Mapping time (min)	Mapping points		LVZ area (${\mathrm{cm}}^{2}$)		CI extent (mm)		Effective ablation site	Effect of ablation on AT
				RA	LA	At default LVT	After LVTA	At default LVT	After LVTA		
1	1	260	18	4326	8227	7.5	0.7	15.8	38	LAPW	Circuit change
2	2	210	12	/	14,788	14.2	0.2	12.5	21.3	LA ridge	Termination
3	3	325	19.7	5027	7081	7.3	1.2	7.1	12.5	LA roof corresponding to BB	Circuit change
4	4	221	16.6	7291	10,227	6.4	0.9	23.7	36	LAAW	Circuit change
5	5	178	10	/	10,839	6.2	0.3	16.1	27.4	MI	Circuit change
6	6	244	16.2	4825	7322	4.6	0.2	14.2	22.6	LAAW	Termination
7	7	255	8	/	13,188	1.6	0.1	11.5	26.5	MI	Termination
8	8	304	11.2	/	9931	7.4	3.5	22.8	30.7	LAAW	Termination
9	9	219	9	/	7065	3.6	0	10.2	41.1	LAAW	Circuit change
	10	235	19	8024	23,705	2.9	0	8.7	23.1	MI	Termination
10	11	310	14.6	/	10,421	8.3	0.2	11.3	26.7	MI	Termination
11	12	263	16.4	6931	15,098	8.6	0.6	17.6	29.9	LAAW	Circuit change
12	13	203	14	4937	8850	5.8	0	18.5	32.9	LAAW	Circuit change
	14	240	12.5	/	13,057	14.7	2.4	7.2	33.1	LAPW	None
13	15	191	9	/	11,465	6.2	0.7	15.5	33.7	LA ridge	Circuit change
14	16	195	15	/	16,178	7.5	1	13.4	21.9	LA roof	Termination

AT, atrial tachycardia; BB, Bachmann’s bundle; CI, critical isthmus; CL, cycle length; LA, left atrial; LAAW, left atrial anterior wall; LAPW, left atrial posterior wall; LVT, low voltage threshold; LVTA, low voltage threshold adjustment; LVZ, low voltage zone; MI, mitral isthmus; RA, right atrial.

**Type 3:**

**Fig. 7.**
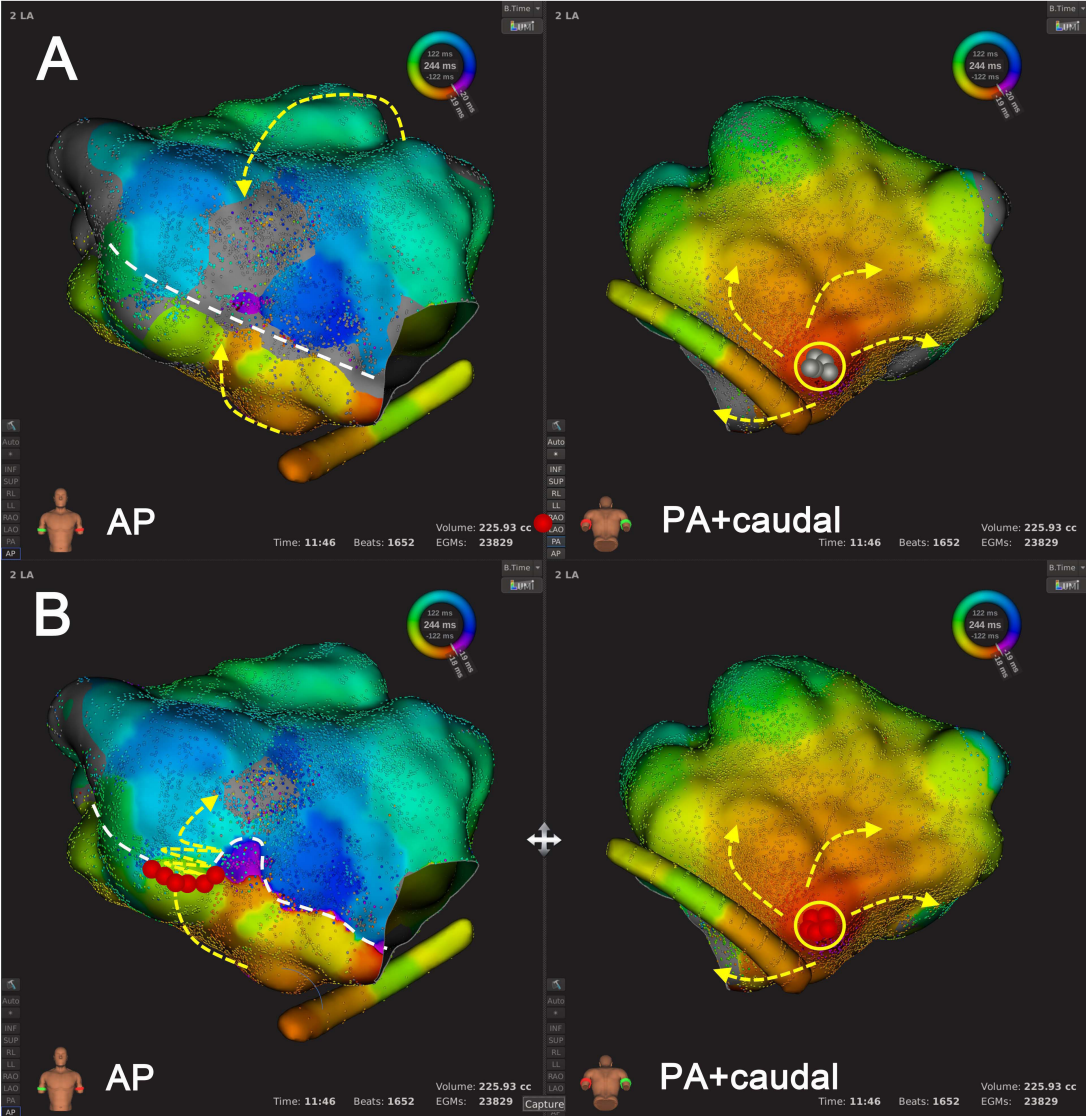
**An example of Type 3 mechanism re-identification**. Panel A shows that at the default LVT of 0.03 mV, activation mapping suggested focal AT originating from LA inferior wall. Panel B shows that after LVTA (0.01 mV), the AT mechanism remained unchanged. However, a conduction gap within the LVZ was revealed following LVTA, through which activation wavefront propagated to activate LA anterior wall. Subsequently, additional ablation was performed to target the conduction gap in the LA anterior wall. Abbreviations: AT, atrial tachycardia; LA, left atrium; LVT, low voltage threshold; LVTA, low voltage threshold adjustment; LVZ, low voltage zone.

In Type 3, the AT circuit and CI remained unchanged, but after adjusting the LVTA to 0.01mV, a bystander conduction gap was discovered (Fig. 7, **Supplementary Videos 11** and **12**). Consequently, after AT termination and sinus rhythm restoration, additional ablation targeting the bystander conduction gap was performed. This type included 3 ATs in 3 patients, and the LVZs were away from AT circuit.

**Fig. 8.**
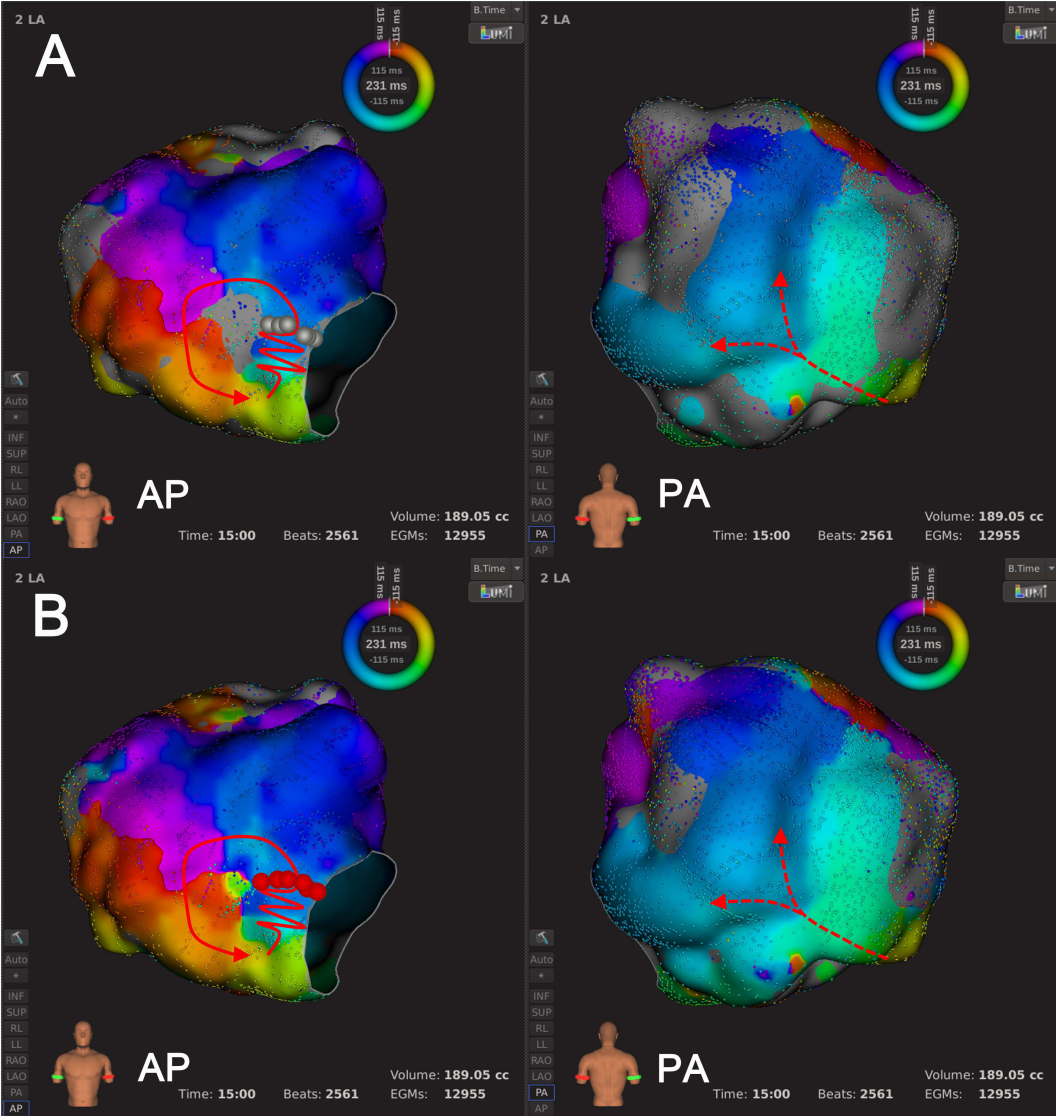
**An example of Type 4.1 mechanism re-identification**. Panel A shows the default LVT (0.03 mV) where activation mapping suggested the wavefront moved counter-clockwise around the LVZ located at LA anterior wall with the LVZ being inside AT circuit. Panel B shows that after LVTA (0.01 mV), the AT circuit remained unchanged. The LVZ was still inside AT circuit and continued to block conduction. Abbreviations: AT, atrial tachycardia; LA, left atrium; LVT, low voltage threshold; LVTA, low voltage threshold adjustment; LVZ, low voltage zone.

**Fig. 9.**
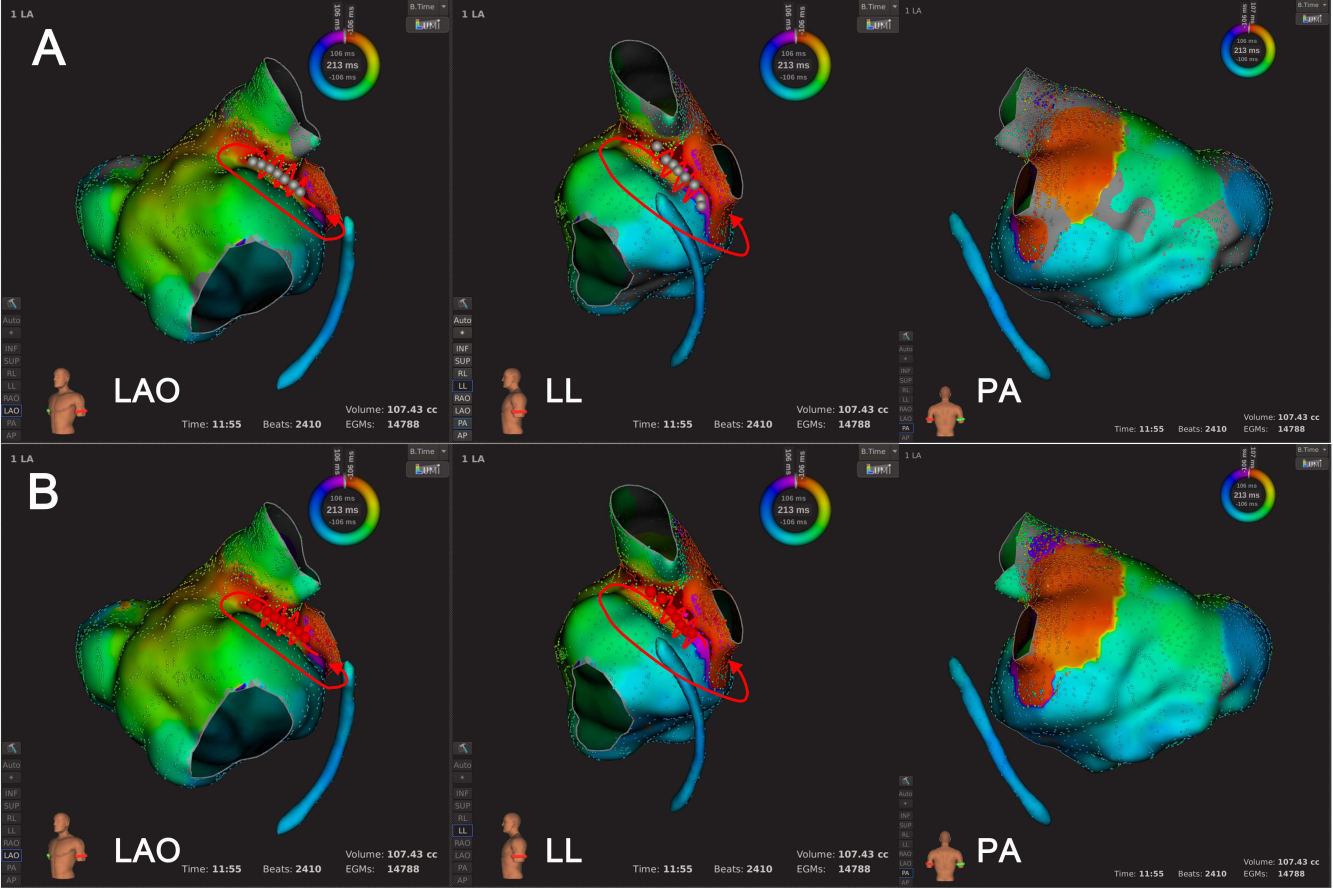
**An example of Type 4.2 mechanism re-identification**. Panel A illustrates that with the default LVT (0.03 mV), activation mapping suggested the wavefront activated the LA ridge in an areal pattern, moved around the base of the LA appendage, following the LA ridge toward the MI. The wavefront then proceeded to activate the LA ridge, possibly through the ligament of Marshall. In this case, the LVZs were not part of AT circuit. Panel B shows that after LVTA (0.01 mV), the AT circuit remained unchanged, but there was a significant decrease in the LVZ in the right area. Abbreviations: AT, atrial tachycardia; LA, left atrium; LVT, low voltage threshold; LVTA, low voltage threshold adjustment; LVZ, low voltage zone; MI, mitral isthmus.

Type 4: After LVTA, AT circuit and CI remained unchanged, and subsequent ablation strategy stayed unaltered. This type included 27 ATs in 20 patients. The following characteristics of LVZ could be observed in this type of AT after LVTA. Type 4.1: complete conduction block was observed within LVZ, regardless of its spatial relation with AT circuit, including 12 ATs in 10 patients (Fig. 8, **Supplementary Videos 13** and **14**). Type 4.2: passive activation propagation could run through LVZ which was located away from the predefined AT circuit at default LVT, including 15 ATs in 11 patients (Fig. 9, **Supplementary Videos 15** and** 16**).

### 3.4 Ablation Results and Follow-Up

Catheter ablation was performed to target the CI for macro-reentrant AT or earliest activation site for focal AT. Effective ablation was achieved in 2 focal ATs, with termination in one AT and circuit change in the other. Out of 53 macro-reentrant ATs, effective ablation was obtained in 48, leading to sinus rhythm restoration in 32 ATs and AT circuit change in the other 16 ATs. In all three Type 3 ATs, additional linear ablation was performed targeting bystander conduction gaps, after which bidirectional block of linear ablation was confirmed.

Catheter ablation was ineffective in treating five macro-reentrant ATs, including one Type 2 AT, one Type 3 AT, and three Type 4 ATs. The reasons for this ineffectiveness included (1) constant changing total CL in 3 ATs, which made mapping unfeasible, and (2) ablation at LA roof where Bachmann’s bundle was distributed with thick myocardium in 2 ATs. Cardioversion was performed to restore sinus rhythm.

At a median follow-up of 16.5 months, the cumulative freedom from AT was 69.3%. After a follow-up of 18.0 ± 9.6, recurrent AT was observed in 16 patients.

## 4. Discussion

The present study reported the results of mapping and ablation of LA tachycardias with a mean LA voltage of less than 0.5 mV using high-density mapping with LVTA. The main findings are as follows. (1) Activation propagation concealed within LVZ was not uncommon, but was typically excluded from activation mapping. (2) By unveiling the activation propagation, LVTA could provide vital information for AT diagnosis. This information could improve the veracity of activation mapping and provide reliable evidence to modify subsequent ablation strategies. (3) The impact of LVTA on AT diagnosis and subsequent ablation could be divided into four categories (a) both AT mechanism and ablation strategy were completely altered ; (b) the AT mechanism remained unchanged and ablation strategy was tailored; (c) a bystander conduction gap was detected; (d) both AT mechanism and ablation strategy remained unchanged.

### 4.1 LVZ and AT Mechanism

Previous studies have examined the characteristics of scar-related ATs from multiple perspectives. For example, Macro-reentrant bi-atrial ATs after AF ablation or cardiac surgery have been reported in multiple studies [3, 13, 14]. Takigawa* et al*. [15] found a close correlation between macro-reentrant AT and ablation-induced LVZ in patients after AF ablation. Tsai* et al*. [16] found that LVA with conduction slowing was potentially predictive of CI for AT. Our previous study also observed that the characteristics of left atrial anterior wall AT correlated with catheter ablation or surgical incision [17]. While the significant role of LVZ as a substrate for AT has been verified, the impact of activation propagation within LVZ on AT mechanism analysis remains unclear.

In our study, 40 patients had a history of cardiac interventions. This included 28 patients (66.7%) treated with radiofrequency catheter ablation and 12 patients (28.6%) who underwent cardiac surgery. Both treatments are capable of inflicting considerable iatrogenic injury to atrial myocardium leading to myocardial scars. The presence of these scars likely explains the extensive low voltage zone and reduced average atrial voltage observed in our study group. In patients with low LA voltage, usually due to physiological atrial remodeling or iatrogenic intervention, conduction challenges can arise. Anatomical structures including the mitral annulus and iatrogenic scars may lead to AT by creating conduction obstacles and slow conduction areas. This complex landscape can make the mapping and analysis of AT a challenging task. Additionally, LVZ may contain bundles of viable myocardium of variable size separated by fibrosis, leading to fractionated, split or late potentials, resembling near-field abnormalities [18]. Differentiating these from BGN can be challenging, resulting in potentially confusing and misleading mapping outcomes. In our study, activation propagation within LVZ was observed in almost half cases (Type 1, 2, and 3), which aided in accurately identifying the true CI and in designing subsequent ablation strategies.

### 4.2 The Significance of LVTA

In the activation map of AT, the points and surrounding areas with bipolar voltage lower than LVT are colored in grey. This coloring is meant to exclude these areas from visualized activation propagation analysis and prevent low-voltage points from affecting the color of activation map. However, such exclusion may result in the loss of crucial information for AT analysis, particularly in patients with an unsatisfactory LA substrate, and therefore may influence the physician’s judgement. Hence, its reasonable to provided sufficiently low and reliable LVT, improving the accuracy of AT activation mapping in this subgroup of patients. This can be achieved through a comprehensive analysis of as many electrograms within the LVZ as possible.

When analyzing AT, LVT plays a critical role in preserving the integrity of the activation mapping. The LVT can be adjusted as needed, but should remain above the BGN level of the mapping system, approximately 0.01 mV for Rhythmia [4]. This setting allows for an extremely low LVT, which preserves the necessary accuracy for timing annotation. Additionally, it avoids introducing artifacts into the map, a factor that is highly beneficial for AT mechanism analysis in patients with extensive LVZ.

### 4.3 The Impact of LVTA on AT Diagnosis and Subsequent Ablation

In our study, we found the use of LVTA significantly enhanced the interpretation of AT mapping in nearly half of AT cases. This included identifying the true AT mechanism (Type 1), tailoring the extent of ablation (Type 2), and uncovering bystander conduction gaps (Type 3). And among all the cases of Type 1 and Type 2 ATs, effective ablation was obtained in all but one instances of Type 2 AT, suggesting the efficacy of LVTA in pinpointing the AT mechanism identification and achieving acute ablation success.

Despite the limited size of our study, the results suggest potential correlations between LVZ location and inadequate AT mechanism analysis. It is suggested that LVTA be conducted under the following conditions. (1) When LVZ is located at inter-atrial connections: if LVZ is found in inter-atrial connections including CS ostium, fossa ovalis, posterior interatrial connections, and LA roof/anterior wall corresponding to distribution of Bachmann’s bundle, there may be a risk of misinterpreting biAT/uniAT. (2) When LVZ overlaps with predefined CI: if LVZ coincides with the predefined CI at corresponding endocardial/epicardial locations, usually at mitral isthmus or LA/RA septum, the possibility of misinterpreting epicardial/non-epicardial AT should be evaluated. (3) When LVZ is adjacent to or inside the predefined AT circuit: in situations where the LVZ is adjacent to or within the predefined AT circuit, potential misinterpretations such as bi-loop/single-loop AT, single loop/focal AT, or single-loop AT, and underestimated CI extent should be considered.

During follow-up, recurrent AT was observed in some patients, highlighting a complex issue that may be attributed to several factors. First, this study included patients with a low mean LA voltage of less than 0.5 mV, suggesting extensive LA fibrosis and areas of slow conduction. Second, rheumatic heart disease was present in almost a quarter of patients, a factor contributing to ongoing cardiac remodeling. Moreover, multiple ATs with different mechanisms were identified in 13 patients, underscoring the complexity of the atrial substrate. Although the follow-up result were acceptable, these findings stress the need for further studies to develop ablation strategies that could reduce the risk of recurrence.

### 4.4 The Advantage of High-Density Mapping

With the help of high-density mapping, AT mechanism analysis could be conducted in a fast and detailed manner, achieving satisfactory accuracy in activation mapping under most conditions. The default LVT settings led to misinterpretations in nearly one-sixth of Type 1 cases with low LA voltage, particularly in patients with extensive LVZ due to the complex AT mechanism.

Accurate identification of unipolar and bipolar signals during AT is the cornerstone of reliable potential measurement and activation timing annotation. This can be challenging when mapping extensively scarred myocardium, resulting in yielding dubious mapping results. By using a basket-shaped mapping catheter with 64 unidirectional electrodes (0.4 ${\mathrm{mm}}^{2}$ and 2.5 mm spacing), the Rhythmia provides a higher resolution and is less influenced by BGN and far-field signals compared to conventional ring-electrodes. The close inter-electrode spacing design also facilitates recordings of a higher bipolar voltage amplitude, improving the signal to noise ratio. Overall, this significantly improves the details of activation mapping, allowing for meticulous illustration in activation mapping interpretation. Furthermore, based on detailed mapping information with adequate accuracy, prudent LVTA can recognize inconspicuous conduction within LVZ by aquiring previously unaccessible voltage data. This extends the comprehension of AT mechanisms and reveals the true CI. Under its guidance, the efficacy of LVTA was partially verified by subsequent effective ablations.

There are several limitations to this study. Firstly, the power of our study is limited by its small sample size, impacting the generalizability of the findings. Secondly, the term ‘low atrial voltage’ remains undefined in this context. While we evaluated the global atrial substrate with mean atrial voltage using an exploratory cutoff value of 0.5 mV, this might not be universally applicable. The data for recorded bipolar voltage may differ depending on the mapping system. Consequently, while the study results are pertinent to the Rhythmia mapping system, extrapolation to other ATs should be done with caution. One of Rhythmia’s strengths lies in the accurate and reliabile identification of unipolar and bipolar potentials. The unidirectional and densely arranged electrodes enable indirect assessment of the catheter contact. However, the catheter doesn’t provide information on contact force, leaving a possibility of low contact during mapping. Our current findings do not fully endorse the benefit of LVTA guided ablation strategy for Type 3 ATs. Further randomized controlled studies are warranted for verification.

## 5. Conclusions

In patients with a poor LA substrate, LVZ is commonly detected during activation mapping, and hidden conduction propagation may frequently be found within it. While this propagation is typically excluded from activation mapping, LVTA can uncover it with dependable accuracy. Overall, this approach enhances the veracity of activation mapping, aiding in the guidance of subsequent ablation.

## Data Availability

The data and materials of this study could be obtained from the corresponding author upon reasonable request.

## Supplementary Material

Supplementary material associated with this article can be found, in the online version, at https://doi.org/10.31083/j.rcm2411329.
