# Supplementary material for: Impact of Low Voltage Threshold Adjustment on Activation Mapping Interpretation for Atrial Tachycardia in Low-Voltage Left Atrium
Source: Rev Cardiovasc Med. 2023 Nov 24;24(11):329. doi: 10.31083/j.rcm2411329 (PMC11272877; doi:10.31083/j.rcm2411329)
Supplement: Supplementary file 1 [file 2153-8174-24-11-329-s1.zip › Supplementary material.pdf]

Supplementary materials

Supplementary methods

High-density mapping and ablation procedure

Before the procedure, transesophageal echocardiography was performed to exclude LA thrombus. Under local anesthesia, a 6F decapolar diagnostic catheter was placed in the coronary sinus (CS) via the femoral vein access as a stable reference for pacing and local activation time annotation. If the patient was in sinus rhythm (SR) before mapping, programmed CS pacing was performed to induce AT. Heparin (100UI/kg) was used to maintain an active clotting time between 300 and 350s.

The IntellaMap Orion catheter, a 64-pole mini-basket mapping catheter, was introduced via a 8.5F Swartz sheath for electroanatomic mapping guided by the Rhythmia system. The atrial chamber surface geometry was generated and continuously updated using the location of the outermost electrodes under respiratory and cardiac cycle gating. Unipolar and bipolar EGM were combined to obtain accurate annotation of the local activation time of each bipolar EGM. And for fragmented or multiple potential EGM, the timing in the surrounding area was used to select potential for timing annotation.

Right atrial (RA) mapping was conducted first if the atrial wave was preceding in proximal CS leads, while left atrial (LA) mapping was performed via transseptal approach if the atrial wave was preceding in distal CS leads or RA mapping results

suggested LA-originated AT, including (1) a missing TCL of >10%; (2) RA activated in a centrifugal pattern and earliest at RA septum.

During mapping, an activation map was created under standard automatic beat acceptance criteria as follows:(1) (1) cycle length (CL) variation <10ms; (2) activation time difference between two CS electrograms ≤5ms; (3) catheter motion ≤1mm during the cardiac circle; (4) the distance between anatomical shell and electrode ≤2mm. Pre-existing linear lesions were identified by conduction block of lines shown as sharp changes in activation time and color on the activation map. The CI was defined as the narrowest part of AT circuit with a slow conduction area formed by conduction barriers on both sides, which was demonstrated as a decrease in the activation area within the AT cycle, as illustrated by Lumipoint™, and further identified by the Skyline tool (Boston Scientific, MA).

The mechanism of each AT was then analyzed by activation mapping result to identify the critical area. An irrigated ablation catheter (Boston Scientific, MA) was used for ablation by targeting the critical area (43℃, 35W, irrigation rate 12ml/min).

References

1. Wang H, Xi S, Chen J et al. Left Atrial Anterior Wall Scar-Related Atrial Tachycardia in Patients after Catheter Ablation or Cardiac Surgery: Electrophysiological

Characteristics and Ablation Strategy. *Journal of cardiovascular development and disease* 2022;9.

Supplementary video legends

Supplementary video 1 and **Supplementary video 2** An example of Type 1.1 AT mechanism re-identification. These two videos illustrated AT mechanism as yielded by activation mapping with LVT at 0.03mV and 0.01mV, respectively.

Supplementary video 3 and **Supplementary video 4** An example of Type 1.2 AT mechanism re-identification. These two videos illustrated AT mechanism as yielded by activation mapping with LVT at 0.03mV and 0.01mV, respectively.

Supplementary video 5 and **Supplementary video 6** An example of Type 1.3 AT mechanism re-identification. These two videos illustrated AT mechanism as yielded by activation mapping with LVT at 0.03mV and 0.01mV, respectively.

Supplementary video 7 and **Supplementary video 8** An example of Type 1.4 AT mechanism re-identification. These two videos illustrated AT mechanism as yielded by activation mapping with LVT at 0.03mV and 0.01mV, respectively.

Supplementary video 9 and **Supplementary video 10** An example of Type 2 AT mechanism re-identification. These two videos illustrated AT mechanism as yielded by activation mapping with LVT at 0.03mV and 0.01mV, respectively.

Supplementary video 11 and **Supplementary video 12** An example of Type 3 AT mechanism re-identification. These two videos illustrated AT mechanism as yielded by activation mapping with LVT at 0.03mV and 0.01mV, respectively.

Supplementary video 13 and **Supplementary video 14** An example of Type 4.1 AT mechanism re-identification. These two videos illustrated AT mechanism as yielded by activation mapping with LVT at 0.03mV and 0.01mV, respectively.

Supplementary video 15 and **Supplementary video 16** An example of Type 4.2 AT mechanism re-identification. These two videos illustrated AT mechanism as yielded by activation mapping with LVT at 0.03mV and 0.01mV, respectively.
